# Shared Decision Making in mental health care using Routine Outcome Monitoring as a source of information: a cluster randomised controlled trial

**DOI:** 10.1186/s12888-015-0696-2

**Published:** 2015-12-15

**Authors:** Margot J. Metz, Gerdien C. Franx, Marjolein A. Veerbeek, Edwin de Beurs, Christina M. van der Feltz-Cornelis, Aartjan T. F. Beekman

**Affiliations:** Trimbos-institute and GGz Breburg, Postbus 770, 5000 AT Tilburg, The Netherlands; Trimbos-institute and Foundation 113Online, Postbus 22232, 1100 CE Amsterdam, The Netherlands; Trimbos-institute, Postbus 725, 3500 AS Utrecht, The Netherlands; University of Leiden and Stichting Benchmark GGZ, Rembrandtlaan 46, 3723 BK Bilthoven, The Netherlands; Tilburg University and Clinical Centre of Excellence for Body, Mind and Health, GGz Breburg, Postbus 770, 5000 AT Tilburg, The Netherlands; VUMC and GGZ inGeest, A.J. Ernststraat 1187, 1081 HL Amsterdam, The Netherlands

**Keywords:** Routine Outcome Monitoring, Shared Decision Making, Quality Improvement Collaborative (QIC), Cluster randomised controlled trial, Mental health care

## Abstract

**Background:**

Shared Decision Making (SDM) is a way to empower patients when decisions are made about treatment. In order to be effective agents in this process, patients need access to information of good quality. Routine Outcome Monitoring (ROM) may provide such information and therefore may be a key element in SDM. This trial tests the effectiveness of SDM using ROM, primarily aiming to diminish decisional conflict of the patient while making decisions about treatment. The degree of decisional conflict, the primary outcome of this study, encompasses personal certainty about choosing an appropriate treatment, information about options, clarification of patient values, support from others and patients experience of an effective decision making process. Secondary outcomes of the study focus on the working alliance between patient and clinician, adherence to treatment, and clinical outcome and quality of life.

**Methods/Design:**

This article presents the study protocol of a multi-centre two-arm cluster randomised controlled trial (RCT). The research is conducted in Dutch specialised mental health care teams participating in the ROM Quality Improvement Collaborative (QIC), which aims to implement ROM in daily clinical practice. In the intervention teams, ROM is used as a source of information during the SDM process between the patient and clinician. Control teams receive no specific SDM or ROM instructions and apply decision making as usual. Randomisation is conducted at the level of the participating teams within the mental health organisations. A total of 12 teams from 4 organisations and 364 patients participate in the study. Prior to data collection, the intervention teams are trained to use ROM during the SDM process. Data collection will be at baseline, and at 3 and 6 months after inclusion of the patient. Control teams will implement the SDM and ROM model after completion of the study.

**Discussion:**

This study will provide useful information about the effectiveness of ROM within a SDM framework. Furthermore, with practical guidelines this study may contribute to the implementation of SDM using ROM in mental health care. Reporting of the results is expected from December 2016 onwards.

**Trial registration:**

Dutch trial register: TC5262

Trial registration date: 24th of June 2015

## Background

Shared Decision Making (SDM) is defined as an approach where clinicians and patients share the best available information when making clinical decisions, and where patients are supported to consider options to achieve informed preferences [1]. An important principle of Shared Decision Making is that there are at least two experts: the patient and the clinician. The process of SDM bridges both expert domains [2, 3]. SDM is seen as an intermediate stage between two extremes a traditional paternalistic and an informed choice model. In SDM the clinician and patient deliberate about treatment options and preferences. They select together the best suitable option which is consistent with patients’ values and preferences [2, 4, 5]. Elwyn et al. [1] describe three steps of SDM: 1) making sure that patients know that reasonable options are available 2) providing detailed information about these options, and 3) supporting the consideration of preferences and decision making about what will be the best choice.

A few studies testing SDM have been conducted in mental health care [2, 6], including a small number of randomised controlled trials and other types of studies, mostly focusing on psychosis and depression. They indicate that SDM contributes to better informed patients regarding the disease and treatment options. Furthermore, it stimulates patients to take an active part in decisions on treatment options. Also, SDM can have a positive influence on patient satisfaction, better acceptance of the agreed treatment, adherence to treatment and reduction of uncertainty by the patient about the decision taken [2, 7–13]. Finally, SDM increases the likelihood that treatment is delivered in accordance with guidelines. Few studies that tested this have found limited effects of SDM on reduction of symptoms [7, 9], mainly by the mediating variable treatment adherence on clinical outcome [9]. Despite the limited but promising results of SDM widespread implementation seems to be lagging behind [2, 14–16], while generally patients prefer greater participation than they are offered [13, 17, 18]. Patients with severe mental illness and depression have positive attitudes towards SDM, desire to be involved in decisions, and are also able to participate in decision making [10, 19–21].

ROM implies regular measurement of clinical outcomes aiming to improve quality of care. ROM provides clinical feedback as to the effectiveness of care for patients and clinicians which may be used to choose appropriate treatment options [22–27].

In a review Carlier et al. [28] found that feedback with ROM has positive effects on the behaviour of professionals with respect to faster and more appropriately screening to establish the correct diagnosis, more frequent and effective communication between patient and clinician, and, when appropriate, swifter adjustment of treatment. ROM appears to have a positive short term effect on mental health status [29] and, if ROM feedback was used in consultations, is especially beneficial for the monitoring of patients who are not responding to treatment, the so called Not-On-Track (NOT) patients [22, 23, 29–32]. The greatest reduction of symptoms takes place in the condition where both parties, patients and clinicians, were provided with feedback [22, 23]. Measuring outcome of patients on a regular basis during treatment will allow clinicians to identify non-responding and negatively responding patients before they leave their treatment. The sooner a clinician can be notified that a patient’s positive outcome is threatened, the sooner preventive actions can be attempted [33].

### Rationale

Both SDM and ROM are able to empower the patient during the treatment process and to provide good quality information in order to be a more effective agent in the decision making process. Such an approach was evaluated in an open pilot in a centre for patients with combined physical and mental disorders. The SDM approach during intake and treatment was combined with frequent ROM. In line with treatment goals, clinician and patient choose symptoms to be monitored with ROM during treatment. The progress in treatment is evaluated with six weekly monitoring used in a feedback cycle. This approach proves feasible, adherence of both patients and professionals is high and patients improve at symptom level. However, so far, a RCT evaluating such a combined SDM-ROM approach has not yet been performed [34]. The expectation is that systematically sharing the feedback about treatment progress derived from ROM within a SDM framework may help to empower patients, improve their involvement in clinical decision making and thereby enhance the quality of care. The SDM-ROM model should be applicable to different subgroups in mental health care.

### Aim and hypotheses

Our aim is to test whether the appliance of a model in which ROM and SDM are integrated is effective compared to decision making as usual where SDM using ROM is not implemented.

The first hypothesis is that the combined SDM-ROM model will lead to less decisional conflict experienced by patients which encompasses more personal certainty about choosing an appropriate treatment, good information about options, clarification of values, support from others and experience of an effective decision making process. Second hypothesis is that the application of the SDM-ROM model will improve the working relationship between patient and clinician, will increase patient’s adherence to treatment and will have positive effects on clinical outcome and quality of life.

## Methods/Design

### Trial design

The study is designed as a multi-centre two-arm cluster randomised controlled trial of Shared Decision Making (SDM) in specialised mental health care using Routine Outcome Monitoring (ROM) as a source of information. A cluster randomised design in which the unit of randomisation is the treatment team is deemed necessary to prevent spill-over effects between intervention and control groups. During recruitment of teams for the study, pairs of teams from the same mental health organisation are randomly assigned to either the experimental or control conditions (matched pairs). Control teams will implement the SDM and ROM model after the completion of the study.

### Participants and setting

The study takes place in specialised mental health care organisations. Due to the expected generic applicability of the SDM-ROM model, the model is being tested to various subgroups with the following characteristics: age (adolescents, adults and elderly patients), diagnosis (psychotic, common mental and personality disorders) and setting (outpatient, day-clinic and clinic). In this study, a total of six intervention and six control teams from four organisations participate. In accordance to the sample size calculation (ref. section sample size) the teams will include 364 patients (182 in the intervention group and 182 in the control condition, with an average of 31 patients per team). Patients who don’t speak and read Dutch are excluded from the study.

### Randomisation and blinding

The matched teams treat a similar population of patients, in a similar geographic catchment area in the Netherlands and have similar personnel. Randomisation of the teams is conducted through an independent data manager. Due to the randomisation at the team level and the nature of the intervention, blinding of the clinicians and patients is not possible. To avoid bias due to lack of blinding outcome parameters will be collected, independent of the research team, with self-report instruments to be completed both by patients and clinicians.

### Intervention

The intervention teams implement the SDM-ROM model with five steps (Table 1) both in the intake and treatment process.

**Table 1:** SDM-ROM model

1. Introduction	➣ Refer to expectations about shared process.
	➣ Discuss which role the patient desires in decision making.
	➣ Connect with patient’s wishes and goals. ‘What does he/she want to achieve in treatment?’
	➣ Explain about ROM as an information source.
2. Give meaning to ROM	➣ Discuss ROM outcomes
	➣ Steps:
	Identify, Understand, Appreciate, Act.
3. Explore options	➣ Discuss options, advantages and disadvantages, in a neutral manner.
4. Weigh options	➣ Weigh advantages and disadvantages: ‘What’s for you important?’
5. Shared Decision	➣ Can a choice be made?
	➣ Select together most appropriate option.
	➣ Make follow-up appointments.

Shared Decision Making using Routine Outcome Monitoring as an information source (Step 1 to 5)

In order to ensure that the use of ROM by clinician and patient has added value in treatment, the intervention teams measure with appropriate questionnaires or rating scales at suitable moments minimal at the beginning, during and at the end of treatment. The type of ROM-instruments and frequency of measuring is not centrally prescribed, but in each intervention team tailored to the patient group. The appliance of the five steps SDM-ROM model is the key intervention. To implement the SDM-ROM model, the intervention teams follow a Quality Improvement Collaborative (QIC) program of 1 year, which can be considered as a multifaceted implementation strategy [35–37], consisting of a mix of improvement strategies, in which training and coaching in the SDM-ROM model are important components:

National:

- Three conference days for the intervention teams for exchange and learning, with the expert team and advisors from the national collaborative support organisation (Trimbos-institute, Netherlands Institute of Mental Health and Addiction, https://www.trimbos.nl/) present.
- Training and booster sessions for the clinicians participating in the intervention teams about the SDM-ROM model.
- A workshop about how to use ROM-instruments.
- A network of multidisciplinary teams.
- Experts providing supervision and advice about ROM best practices.
- Meetings between local team coordinators for exchange and learning, with the Trimbos-institute advisors present.
- Meetings for local patient representatives for exchange and learning.
- A QIC ROM website for exchange of presentations, publications, best practices and tools.

Local:

- Team visits and telephone contacts with Trimbos-institute advisors for advice and coaching.
- SMART goal setting, actions and indicators written in action plans (PDCA) by the local teams. Written feedback on local action plans by Trimbos-institute advisors.
- Teams organise meetings at their own location to work on their improvement plans. The teams plan, implement, evaluate and adjust their actions to improve the application of ROM as a tool in SDM (PDCA-cycle).
- Feedback to the local teams with a general survey about ROM in daily clinical practice (Trimbos-institute, 2015).
- Involvement of patient representatives in improvement teams.
- Active management involvement with the intervention teams.

### Data collection

#### Procedures

To prevent selection bias, clinicians in both groups invite all patients, based on date of the first treatment session, to participate in the study during a period of 6 months. In long term treatment teams, patients are selected by chronological order of treatment evaluation date. This group is also followed for a period of 6 months.

The clinicians of the intervention and control teams inform their patients about the study and obtain informed consent.

In case a patient is transferred to another clinician within the intervention team during the course of the study, the SDM and ROM process is transferred to this clinician. If the patient is transferred to another team during the treatment, for example from the intervention to the control team or vice versa, the patient can no longer participate in the study.

Data collection is conducted by independent research assistants outside the participating organisations. Depending on the frequency of consultations between clinician and patient using the SDM-ROM model, patients are completing self-report questionnaires at three times (baseline = T0, 3 months after baseline = T1, and 6 months after baseline = T2) or twice (baseline = T0 and 6 months after baseline = T2). In both frequencies of measurement the questionnaires for the study are sent after the SDM-ROM conversation. In addition, clinicians answer questions at T2 regarding their patients. Patients and clinicians who participate in the study receive a link by email to complete the questionnaires. If necessary, 1 week later they get a reminder by email. After 2 weeks an assistant phones the patients who have not yet answered the questions. If patients do not use internet, they receive paper questionnaires by mail.

#### Outcome measures

##### Primary outcome measure

**Fig. 1:**
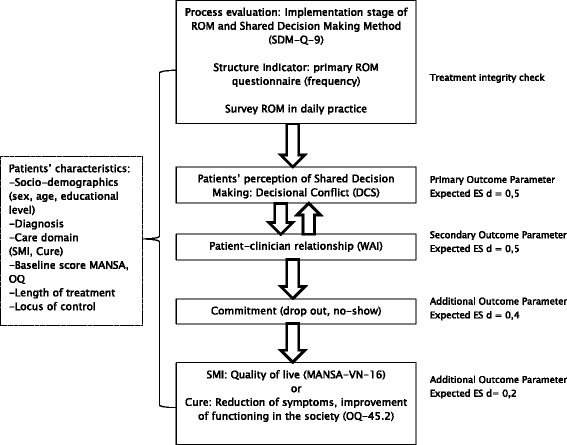
Primary and secondary outcome parameters in flow chart

The primary outcome measure (Table 2, Fig. 1) is the degree of decisional conflict, a central determinant of decision making, which is defined as ‘personal uncertainty about which option to choose’ [38]. This will be measured with the translated Decisional Conflict Scale (DCS). The DCS is a self-report questionnaire comprising sixteen questions about personal perceptions of: a) uncertainty in choosing options, b) modifiable factors contributing to uncertainty such as feeling uninformed about alternatives, benefits and risks, unclarity about personal values and unsupported in decision making, c) effective decision making such as feeling the choice is informed, values-based, likely to be implemented and expressing satisfaction with the choice [39]. Each item is measured on a 5-point Likert scale (0 = strongly agree to 4 = strongly disagree). Besides the total score, the DCS has the following five sub scores: certainty, information, clarification of values, support or pressure from others and the patient’s perception of the quality of the decision process. To calculate the total and sub scores the item scores will be summed, divided by the number of items and multiplied by 25. The scores thus range from 0 (no decisional conflict) to 100 (extremely high decisional conflict). By appropriate application of SDM using ROM, decisional conflict is expected to be lowered. Individuals whose scores will be higher than 37.5, are considered to be uncomfortable with the decision and tend to delay it. Scores lower than 25 will be associated with absence of decisional conflict. In this study we will examine whether the total and sub scores at this scale are associated with the implementation of the SDM-ROM model [38, 39].

**Table 2:** Primary and secondary outcome parameters

Topic & Instrument	Method	Patient	Patient	Patient	Clinician	Organisation
		T0	T1	T2		
Primary Outcome Parameter						
Patient’s perception of SDM						
Patient						
Decisional Conflict Scale	SR	X	X	X		
Secondary Outcome Parameter						
Patient-clinician relationship						
Patient						
Working Alliance Inventory	SR	X	X	X		
Clinician						
Working Alliance Inventory (patient level)	SR				X (T2)	
Additional outcome measures						
Compliance						
Organization						
Drop out (patient level)	EPR					X (T2)
No-Show (patient level)	EPR					X (T2)
Symptoms-functioning						
Patient						
OQ-45	SR	X	X	X		
Quality of life						
Patient						
MANSA-16	SR	X	X	X		

*SR* self-report, *EPR* Electronical Patient Record

T0 = baseline

T1 = Follow-up +/−3 months after baseline (between 10 and 16 weeks)

T2 = +/−6 months after baseline (between 23 and 29 weeks)

The psychometric properties of the scale are acceptable. Test-retest correlations and Cronbach alpha coefficients exceed 0.78 (reliability). The scale correlates to related constructs (knowledge, regret, discontinuance) and discriminates between groups who make and delay decisions (construct validity). DCS is responsive to change between different decision supporting interventions. Effect sizes range from 0.4 to 1.2 for the total scale. The predictive validity of the DCS is also demonstrated. Finally, the instrument is easy to administer [38].

Application of the revised Dutch DCS is not known to us. For this study the DCS is translated from English into Dutch by two translators separately, who subsequently established a consensus version. The consensus version was back translated into English by one independent native English translator. The native speaker and the research team checked and discussed the discrepancies between the original version and the translation, which resulted in a final Dutch version [40].

#### Secondary outcome measures

The secondary outcome measures (Table 2, Fig. 1) are the patient-clinician relationship, patient’s adherence to treatment, clinical outcome and quality of life. The patient-clinician relationship will be assessed using the Dutch version of the Working Alliance Inventory Short Form (WAI-S) [41–43]. For this study the WAI-S is chosen, because the questionnaire measures the quality of the alliance on the basis of three aspects related to SDM: contact/bond, agreement on goals and agreement on tasks [43–45]. Besides, the WAI-S has patient and clinician versions, which are both used in this study. The questionnaire consists of twelve questions concerning the agreement about treatment targets and tasks, and the interpersonal connection between patients and clinicians. The WAI-S scores from 1 (rarely or never) to 4 (always). Stinckens et al. [43] report a three factor structure of the patient version of the instrument: a contact/bond, a task and a goal component. Tracey et al. [41] also found evidence for one overall score. Psychometric qualities of the WAI-S proved to be good [41,43, 46, 47]. The scale correlates with outcome moderately. The perspective of the patient and a high score on the task scale are the strongest predictors for positive outcome [43].

In addition to these outcomes, the influence of the patients’ commitment to the treatment plan will be investigated. Data about no-show and drop out will be extracted from the electronic patient record by each team.

Finally, effects of SDM using ROM on treatment results will be studied with either the Manchester Short Quality of Live Measurement (MANSA-VN-16) [48] for long term patients or the Outcome Questionnaire (OQ-45) [49] for short term patients.

The MANSA-VN-16 is a self-report questionnaire which measures how satisfied the patient is in each of the following life domains: living situation, social relationships, physical health, mental health, safety, financial situation, work situation and life as a whole. Twelve questions are answered on a 7-point scale (1 = not satisfied, 7 = very satisfied) and four items are rated by ‘yes’ or ‘no’. An overall score of subjective quality of life can be calculated as a mean overall score. A higher score on the MANSA means a better quality of life. The psychometric properties are satisfactory [50].

De OQ-45 is a self-report scale with 45 items, which measures symptoms, emotional states, interpersonal relationships and social role functioning. Each item is measured on a 5-point Likert scale (0 = never to 4 = almost always). Scoring is reversed for nine positively formulated items. The scores of the items are summed. This means a total score ranging from 0 to 180. Higher scores reveal reporting of more frequent symptoms, distress, interpersonal problems and social dysfunction. Apart from the total scale, the Dutch OQ-45 has the following four subscales: symptomatic distress, anxiety and somatic distress, interpersonal relations, social role. Results show that the psychometric properties of the Dutch OQ were adequate and similar to the original instrument [51].

#### Treatment integrity

To check the treatment integrity in the intervention teams, one structure indicator will be measured and three process evaluation approaches will be conducted (Table 3). The structure indicator will report how frequently the primary ROM-instrument is completed. The process evaluation will indicate the implementation stage of the SDM-ROM model in the intervention teams.

**Table 3:** Treatment integrity check and measuring moments

Topic & Instrument	Method	Patient	Patient	Patient	Clinician
		T0	T1	T2	
Treatment Integrity Check					
Patient					
SDM-Q-9	SR	X	X	X	
ROM-questions	SR	X	X	X	
Clinician					
SDM-Q-9 (patient level)	SR				X (T2)
ROM-questions (patient level)	SR				X (T2)
Survey about ROM in daily clinical practice. (clinician level)	SR				X (T0, T1, T2)

T0 = baseline

T1 = Follow-up +/−3 months after baseline (between 10 and 16 weeks)

T2 = +/−6 months after baseline (between 23 and 29 weeks)

Survey ROM in daily practice in intervention and control group

The following three process scales will be used:

1. Four questions for patients and clinicians, developed by the research team, about the added value and use of ROM feedback in the conversation between patients and clinicians. Patients in the intervention group will be asked to fill out the four ROM-questions at T0, T1 and T2. At T2 clinicians will complete the same questions regarding the consultations with their patients.
2. The SDM-steps will be measured with the translated Shared Decision Making-Questionnaire-9 (SDM-Q-9), which has a version for patients and one for clinicians. Both versions ask the patient and clinician first to enter the health problem the consultation was about and which decision was made. The questionnaire continues with nine items about the steps in the SDM process, scoring at a six point scale that ranges from 0 (completely disagree) to 5 (completely agree). A total score can be calculated by summing the scores of all items. A high score indicates more SDM. The SDM-Q-9 shows a high reliability. The available results about the factorial validity are also positive [51, 52]. The SDM-Q-9 versions were translated into Dutch by the research team conform the standards of forward and backward translations (ref. section primary outcome measure, [40]). A recent publication about psychometric testing of a translated Dutch version of the SDM-Q-9 [53] demonstrated good acceptance, internal consistency, and acceptable to good convergent validity of the versions for patients and physicians. Patients in the intervention group will be asked to complete the SDM-Q-9 at T0, T1 and T2. At T2 clinicians will complete the same questions regarding the consultations with their patients.
3. A general survey for clinicians of the intervention and control group at three moments in a year about ROM in daily clinical practice [50]. The survey consists of one question about the extent of using ROM in daily practice, with five response categories from ‘never’ to ‘always’. If a clinician scores ‘never’, the first question is followed by an open question about the reason(s) of this. After scoring the other answers, 23 statements about the implementation of using ROM will be submitted. The statements have five response categories, ranging from ‘strongly disagree’ (score 1) to ‘strongly agree’ (score 5). A higher score means a better implementation of ROM in daily practice. The development of the survey is described in a paper of the Trimbos-institute, Netherlands Institute of Mental Health and Addiction [54].

#### Patients’ characteristics at baseline: moderator and potential covariates

At baseline the following characteristics of patients (Table 4) will be collected: socio-demographics (sex, age, and educational level), diagnosis, length of treatment and locus of control. Locus of control is measured with the Mastery Scale [55]. This scale is designed to measure the degree to which people perceive that they can control factors that influence their life situation. The scale has been widely used and translated into multiple languages. Because no standardized scoring recommendations were provided, investigators developed their own scoring protocols and the studies are difficult to compare. The original Pearlin Mastery Scale showed good construct, predictive validity and internal consistency. The Dutch version, used in the baseline measurement of this study, consists of five negatively worded items with five ordered response categories from 1 (strongly agree) to 5 (strongly disagree), where 5 indicates the highest level of self-mastery. A total score can be calculated by summing the scores of all items (range 5–25). This version is also used in NEMESIS-I, a Dutch study of mental and physical health and wellbeing. The reliability of this version is good [55].

**Table 4:** Patients’ characteristics

Topic & Instrument	Method	Patient	Clinician	Organisation
		T0	T0	T0
Patients’ characteristics at baseline				
Patient				
Educational level	SR	X		
Locus of Control-Mastery	SR	X		
Organisation				
Age	EPR			X
Sex	EPR			X
Diagnosis	EPR			X
Care domain	EPR			X
Length of treatment	EPR			X

*SR* self-report, *EPR* Electronical Patient Record

### Sample size

This study is designed to detect a medium effect size of *d* = 0.5 on the primary outcome between the intervention and control group. A significance level set at α = 0.05 and 65 patients per arm yield a power of 0.80 [56]. Due to the cluster-randomisation at team level, we will calculate the effective sample size with an intra cluster correlation coefficient (ICC). The ICC is a measure of relatedness of responses within a cluster [57]. When adjusting for clustering within teams we expect an ICC = 0.05. Using the following formula [57]: Design Effect (DE) = 1 + (m-1) ICC (0.05), the sample size needs to be 136 patients per arm. Finally we expect a drop out of 25 %. This requires an initial inclusion of 182 patients per arm, which means that on average 31 patients per team per arm, will be included.

### Statistical analyses

The data will be analysed according to the intention-to-treat principle. In addition, a completer analysis will be performed. In order to assess whether randomization resulted in similar groups, differences between the two arms will be examined on relevant variables (e.g. sex, age, educational level, diagnosis, length of treatment, locus of control) using independent samples t-tests for the continuous variables and Chi-Square tests for the categorical variables. Variables that are distributed unevenly among the two arms will be entered as covariates when testing the effectiveness of the intervention. The hypotheses will be tested using multivariate linear regression and, due to the cluster randomisation, multi-level analyses.

In addition, the patients’ and clinicians’ views on the ROM-application (ROM questions), Shared Decision Making process (SDM-Q-9) and Working Alliance (WAI) will be compared. If they show different views, the interaction with the primary and secondary outcome parameters will be studied.

Analyses will be conducted using SPSS. Reporting of the results of the study will be in accordance with the CONSORT statement 2010 (extension cluster randomised trials).

### Ethics and trial registration

The Medical Ethics Review Committee of VU University Medical Centre declared that the Medical Research Involving Human Subjects Act (WMO) does not apply to this study (reference number: 2015.237). An official approval of this study by our committee is not required. Patients will be informed of all procedures and asked for written informed consent. The patients will be informed that they can withdraw their consent to participate at any time without specification of reasons and without negative consequences with regard to future treatment. This study is registered in the Dutch Trial Register with number: TC5262, registration date: 24th of June 2015.

## Discussion

Although previous results of SDM in mental health care are promising, few studies have been conducted in this sector [2, 6]. Integrating systematic clinical feedback derived from ROM within a SDM framework may further enhance the communication between patient and clinician, empowerment of the patient and lead to better treatment outcomes. To investigate SDM in mental health care using ROM, a multi-centre two-arm cluster randomised controlled trial will be conducted, which hypothesizes positive effects on 1) patient’s perception of decisional conflict, 2) the working alliance between patient and clinician, 3) adherence to treatment, and 4) clinical outcome and quality of life.

Strength of this study is the matched pair design. Due to randomisation at cluster level between two similar teams of the same organisation, the risk of confounding is reduced. Second, a strong aspect is that the data collection for this research, with self-report instruments to be completed both by patients and clinicians, is conducted through independent research assistants, which diminishes undesired influence of the research team or clinicians on the results. The independent data collection also reduces the chance of social desirable answers and enhances uniformity and quality of the collected data.

The third strength of the study is that at T2, both the patients and the clinicians of the intervention group are invited to complete questionnaires about the use of ROM in the SDM framework. In addition, in both conditions, the working alliance between patient and clinician is examined from both points of view. If there are different views between patients and clinicians, they will be detected and further analysed.

Finally, as this study is conducted in real world clinical practices, the study will have good external validity. By its broad scope, including various ages, diagnostic groups and therapeutic settings, the results will be generalizable to a large group of mental health care teams, clinicians and patients.

The study has some limitations that could influence the results. First, the clinicians are not blinded for the design. It is possible that clinicians, working in the control teams, make additional efforts to improve SDM using ROM. In addition, there is a chance that knowledge about SDM and ROM from the intervention group will cross over to the control group. Despite the fixed composition of the teams, there probably will be changes in the participating organisations and in the composition of teams which may affect the study and its findings. If this is the case the principal investigator will deliberate with the project manager and the involved clinicians about the best solution.

If the clinical use of ROM within a SDM framework is found to be effective, the implementation of both ROM and SDM in mental health care will be encouraged. Such a finding will also give the field practical guidance on how to implement SDM using ROM in the best way, which may contribute to the empowerment of patients in mental health care.

## Abbreviations

ROM: Routine Outcome Monitoring
SDM: Shared Decision Making
SDM-ROM model: Shared Decision Making using ROM as a source of information
